# Inequalities in referral pathways for young people accessing secondary mental health services in south east London

**DOI:** 10.1007/s00787-020-01603-7

**Published:** 2020-07-18

**Authors:** Zoe Chui, Billy Gazard, Shirlee MacCrimmon, Hannah Harwood, Johnny Downs, Ioannis Bakolis, Catherine Polling, Rebecca Rhead, Stephani L. Hatch

**Affiliations:** 1 Department of Psychological Medicine, Institute of Psychiatry, Psychology & Neuroscience, King’s College London, London, UK; 2 Department of Child and Adolescent Psychiatry, Institute of Psychiatry, Psychology & Neuroscience, King’s College London, London, UK; 3 Department of Biostatistics & Health Informatics, Institute of Psychiatry, Psychology & Neuroscience, King’s College London, London, UK; 4 Department of Health Service & Population Research, Institute of Psychiatry, Psychology & Neuroscience, King’s College London, London, UK; 5 South London and Maudsley NHS Foundation Trust, London, UK; 6 Economic and Social Research Council (ESRC) Centre for Society and Mental Health, King’s College London, London, UK

**Keywords:** Inequality, Adolescent, Ethnicity, Mental health

## Abstract

Differences in health service use between ethnic groups have been well documented, but little research has been conducted on inequalities in access to mental health services among young people. This study examines inequalities in pathways into care by ethnicity and migration status in 12–29 years old accessing health services in south east London. This study analyses anonymized electronic patient record data for patients aged 12–29 referred to a south east London mental health trust between 2008 and 2016 for an anxiety or non-psychotic depressive disorder (*n* = 18,931). Multinomial regression was used to examine associations between ethnicity, migration status, and both referral source and destination, stratified by age group. Young people in the Black African ethnic group were more likely to be referred from secondary health or social/criminal justice services compared to those in the White British ethnic group; the effect was most pronounced for those aged 16–17 years. Young people in the Black African ethnic group were also significantly more likely to be referred to inpatient and emergency services compared to those in the White British ethnic group. Black individuals living in south east London, particularly those who identify as Black African, are referred to mental health services via more adverse pathways than White individuals. Our findings suggest that inequalities in referral destination may be perpetuated by inequalities generated at the point of access.

**Electronic supplementary material:**

The online version of this article (10.1007/s00787-020-01603-7) contains supplementary material, which is available to authorized users.

## Introduction

In the UK, child and adolescent mental health services (CAMHS) are available for children and young people up to their 18th birthday; and adult mental health services (AMHS) provide specialist care for those aged 18 and over. However, weak institutional and financial linkages between CAMHS and AMHS create gaps in service provision for young people aged 16–17 who are either transitioning from child to adult mental health services or accessing services for the first time [1, 2]. Ages 16–17 are a particularly vulnerable time when the young person is more susceptible to mental illness due to physiological changes and making important decisions about education, employment, housing, and relationships [3]. Despite their vulnerabilities, young people significantly underutilize mental health services, with ethnic minority groups having the greatest unmet need in both the UK and the US [4–8]. Thus, it is important to identify differences in where young people are accessing secondary mental health services (e.g., outpatient, inpatient, or emergency/crisis services) and what role referral source plays in these variations, particularly across the transition between adolescent and adult service provision.

Previous research indicates that ethnicity may be associated with how individuals come into contact with secondary mental health services [9–13]. For example, compared with White adult patients, Black African and Black Caribbean patients in the UK are more likely to access mental health services through the criminal justice system and less often through general practitioner (GP) referral [14]. Furthermore, once referred to secondary health services, some US studies have shown that, compared to a non-Hispanic White group, ethnic minorities are more likely to use inpatient and emergency services [15, 16], and less likely to use outpatient services after adjusting for covariates, such as low socioeconomic status and poor health [16–18]; this underrepresentation of certain ethnic groups in outpatient care appears to play a role in increasing rates of hospitalization and lengths of stay [7, 18]. However, findings are mixed and there is a dearth of UK studies examining referrals to outpatient, inpatient, and emergency services among ethnic minority youth, especially for those aged 16–17 who are at risk of being lost in the service provision gap between child and adult mental health services [19–22]. Moreover, there may be additional barriers to accessing or remaining in touch with mental health services for 16–17 years old from ethnic minority backgrounds such as language barriers, social stigma of mental illness, imbalance of power and authority between service users and providers, and insensitivity and discrimination towards the needs of ethnic minority service users [8, 23, 24]. Migration status and gender have also been overlooked in identifying inequalities in access; there is a lack of data on migrant’s use of health services in the UK [25] and studies examining gender inequalities in access to health care have often focused on physical health services only [26]. Studies that have examined access to mental health services, primarily conducted in the US, found that ethnic minority youth were less likely to have received a diagnosis or treatment for mental health problems compared with White adolescents [27].

### Current study

It is important to identify inequalities in health services that may negatively impact the mental health needs of adolescents, especially during the transition to young adulthood [28]. This paper addresses this gap in the literature by examining inequalities in both referral source and referral destination for young people aged 12–29 accessing secondary mental health services in south east London. Using a large population sample of service users, the study aimed to (i) identify inequalities in referral source by age, ethnicity, migration status, and gender, (ii) examine differences in referral destination by age, ethnicity, migration status, and gender, and (iii) examine associations between referral source and referral destination. We hypothesized that ethnic minority patients, especially those aged 16–17, would be more likely than White British patients to be (a) referred from social/criminal justice services (i.e., social services, courts, police, probation services, and youth justice services) compared to GP, and (b) referred to inpatient and emergency services (i.e., emergency mental health services and crisis teams) compared to outpatient services.

## Methodology

### Setting and data source

The study was conducted using data from the South London and Maudsley (SLaM) National Health Service (NHS) Foundation Trust Biomedical Research Centre Case Register and the Clinical Record Interactive Search (CRIS) data extraction tool. It provides researcher access to pseudonymised clinical data of over 350,000 patients from SLaM, one of Europe’s largest mental health care providers covering a geographic catchment of 1.3 million residents in four boroughs of south east London (Lambeth, Southwark, Lewisham, and Croydon). This data resource and a comprehensive profile of CAMHS populations within the SLaM catchment area have been described previously [29–33]. CRIS data include structured fields (for demographic, referral source, and referral destination data) and de-identified unstructured free-text fields from case notes and correspondence.

### Participants

The records of all patients with a diagnosis of anxiety or non-psychotic depressive disorder recorded in SLaM from 1st January 2008 to 31st December 2016 and who were aged 12–29 at first referral were retrieved from CRIS (*n* = 21,586). Diagnosis of anxiety or non-psychotic depressive disorder was determined from structured fields in the source record (based on ICD-10 codes F32, F33, F34, F38, F39, F40, F41, F42, F43, F48, F91, F92, and F93) and supplemented by a bespoke natural language-processing application using General Architecture for Text Engineering software [34]. This application derives structured text data from free-text fields in the clinical records (including case notes and correspondence), taking into account the linguistic context and returning strings associated with diagnostic statements [31, 35–37]. Patients who had a co-morbid diagnosis of severe mental illness, for example, schizophrenia spectrum, mania, psychotic depressive, and bipolar affective disorders (i.e., ICD-10 codes F20–29, F30–F31, F32.3, or F33), were excluded due to differences in the identification and clinical response to psychotic disorders in young people compared to non-psychotic affective disorders.

### Outcome variables

Referral source was extracted from structured fields in CRIS and consisted of the following categories: general practice (GP); secondary healthcare services (which included accident and emergency [A&E], clinical specialties, and mental health trusts); and social/criminal justice services (which included both local authority and non-SLaM social services, courts, police, probation services, and youth justice services). Data on referral source were excluded for approximately 23% of the sample: self-referrals (1%) and direct referrals from schools and child health (5%) were excluded due to low cell sizes; referrals from other agencies (< 1%) and any other sources (16%) were excluded, because we were unable to extract the details of the referral source from the database. Referral destination was identified in the CRIS database as the location where patient received care, and categorised as either outpatient, inpatient, or emergency (i.e., emergency mental health services and crisis teams).

### Exposure variables

Year of birth rather than date of birth is retained within the health records as part of the record pseudonymisation process. Age at first referral was, therefore, calculated from year of birth. Patients were divided into three age groups: 12–15, 16–17, and 18–29 years old due to the national legal framework governing the admission to hospital and treatment for those under the age of 18 (classed as a young person) and those aged under 16 (classed as a child) [38–40]. Self-ascribed ethnicity was classified according to the 2011 Census categories [41] and migration status was derived using self-reported country of origin (UK or other). Ethnic groups were collapsed to White British, White Other, Black Caribbean, Black African, Black British, Asian, Mixed, and Other ethnicity due to low numbers in some ethnic groups. For around 2000 people who were assigned to the Black Other group, text was extracted from the free-text fields using a list of search terms that are available from the authors, where possible individuals were then re-assigned to Black African, Black Caribbean, or Mixed ethnicity. Due to over 95% of the Black Other group being non-migrants, the group was re-named to Black British.

### Additional sociodemographic and cohort information

CRIS was used to extract data on gender, household composition, and year of referral. Household composition was self-reported and classified as living with mother, father or both parents, with other family, in foster care, alone, with friends, with a partner, with children only or with others. Household composition groups were collapsed to two categories; living with parents or family and not living with parents or family. Year of referral, extracted using CRIS, ranged from 2008 to 2016.

### Psychosocial functioning

#### Adaptive functioning in those under 18 years old

The Children’s Global Assessment Scale (CGAS), adapted from the Global Assessment Scale for adults, is a rating used to assess functioning aimed at children and young people aged 6–17 years [42]. The child or young person is given a single score between 1 and 100, based on a clinician’s assessment of a range of aspects related to a child’s psychological and social functioning. The score will put them in one of ten categories that range from ‘extremely impaired’ (1–10) to ‘doing very well’ (91–100). Case criteria were defined by a score of 0–70 (case) and 71–100 (non-case).

#### Severity of psychopathology in those aged 18 or older

The Health of the Nation Outcome Scales (HoNOS) is a validated instrument routinely used by professionals to describe health and functioning in individuals with mental health problems [43]. The scale comprises 12 items measuring different aspects of behaviour, impairment, symptoms, and social functioning that are related to illness severity [44, 45]. Each item is rated on a Likert-style scale, ranging from 0 (no problem) to 4 (severe problem). Higher total scores are indicative of more severe psychopathology which was represented using a four-category variable: subclinical (participant scores less than 2 on every item), mild (participant scores 2 on at least one item and less than 2 on all other items), moderately severe (participant scores 3 or more on exactly one item and 2 or less on all other items), and very severe (participant scores 3 or more on at least two items).

### Statistical analysis

Due to the national legal framework governing the admission to hospital and treatment for those under the age of 18 (classed as an adult) and those aged under 16 (classed as a child) [38–40], the sample was divided into three age groups: 12–15, 16–17, and 18–29. Descriptive statistics were calculated to describe the full sample and each of the three age groups (12–15, 16–17, and 18–29) by sociodemographic indicators, referral source, referral destination, year of referral, and psychosocial functioning (Table 1). Inequalities in referral source were examined by ethnicity, migration status, and gender for those aged 12–15, 16–17, and 18–29 by calculating relative risk ratios with 95% confidence intervals with the use of multiple regressions (Table 2). Inequalities in referral destination (inpatient, outpatient, and emergency services) were examined by ethnicity, migration status, and gender for those aged 12–15, 16–17, and 18–29 with the use of multiple regressions (Table 3). To further explore inequalities in referral destination, logistic regressions were conducted to calculate odds ratios with 95% confidence intervals with referral destination as the outcome and referral source, ethnicity, migration status, and gender as the explanatory variables (Table 4). All models were adjusted for sociodemographic indicators (age, ethnicity, migration status, and gender), year of referral, and psychosocial functioning (CGAS score for those aged 12–15 and 16–17; HoNOS score for those aged 18–29). Due to low cell sizes, emergency services were dropped from the analyses, creating a binary variable for referral destination; inpatient services and outpatient services (reference group). High levels of missingness on variables are a limitation of the case registry approach [29]. Multiple imputation by chained equations was performed to address missing data by predicting missing values for any variable from existing values from other variables (for details, see supplementary materials [Table 1]). Ten imputed datasets were created to reduce sampling variability from the imputation process [46]. Odds ratios and relative risk ratios were calculated using the imputed dataset, while frequencies and proportions were calculated using the non-imputed (i.e., original) dataset. All analyses were conducted in Stata 15.1 [47].

**Table 1:** Sample characteristics for full sample and by age group

	Full sample (*n* = 18,931)	Age 12–15 (*n* = 4808)	Age 16–17 (*n* = 2427)	Age 18–29 (11,696)
Age at first episode	Mean 20.3 (S.D 5.3)			
Ethnicity				
White British	8016 (42.3)	2024 (42.1)	923 (38.0)	5069 (43.3)
White other	1581 (8.4)	336 (7.0)	196 (8.1)	1059 (9.1)
Black Caribbean	1071 (5.7)	364 (7.6)	166 (6.8)	541 (4.6)
Black African	1579 (8.3)	356 (7.4)	227 (9.4)	996 (8.5)
Black British	772 (4.1)	300 (6.2)	127 (5.2)	345 (3.0)
Asian	1195 (6.3)	217 (4.5)	167 (6.9)	811 (6.9)
Mixed	983 (5.2)	477 (9.9)	167 (6.9)	339 (2.9)
Other	1870 (9.9)	236 (4.9)	187 (7.7)	1447 (12.4)
Not reported (missing)	1854 (9.8)	498 (10.4)	267 (11.0)	1089 (9.3)
Migration status				
Non-migrant	6058 (32.0)	1197 (24.9)	674 (27.8)	4187 (35.8)
Migrant	2707 (14.3)	255 (5.3)	267 (11.0)	2185 (18.7)
Not reported (missing)	10166 (53.7)	3356 (69.8)	1486 (61.2)	5324 (45.5)
Gender				
Female	11835 (62.5)	3144 (65.4)	1563 (64.4)	7128 (60.9)
Male	7096 (37.5)	1664 (34.6)	864 (35.6)	4568 (39.1)
Referral source				
GP	7391 (39.0)	2227 (46.3)	1032 (42.5)	4132 (35.3)
Secondary health services^a^	6387 (33.7)	698 (14.5)	626 (25.8)	5063 (43.3)
Social/criminal justice^b^	771 (4.1)	302 (6.3)	203 (8.4)	266 (2.3)
Not reported (missing)	4382 (23.2)	1581 (32.9)	566 (23.3)	2235 (19.1)
Referral destination				
Outpatient	12226 (64.6)	4233 (88.0)	1817 (74.9)	6176 (52.8)
Inpatient	1475 (7.8)	257 (5.4)	188 (7.8)	1030 (8.8)
Emergency	4745 (25.1)	214 (4.5)	361 (14.9)	4170 (35.7)
Not reported (missing)	485 (2.6)	104 (2.2)	61 (2.5)	320 (2.7)
Household composition				
Living with parents/family	5712 (30.2)	2542 (52.9)	1025 (42.2)	2145 (18.3)
Not living with parents/family	4660 (24.6)	215 (4.5)	282 (11.6)	4163 (35.6)
Not reported (missing)	8559 (45.2)	2051 (42.7)	1120 (46.2)	5388 (46.1)
Year of referral				
2008	2300 (12.2)	625 (13.0)	282 (11.6)	1393 (11.9)
2009	2408 (12.7)	694 (14.4)	303 (12.5)	1411 (12.1)
2010	2466 (13.0)	611 (12.7)	332 (13.7)	1523 (13.0)
2011	2146 (11.3)	539 (11.2)	282 (11.6)	1325 (11.3)
2012	2031 (10.7)	536 (11.2)	254 (10.5)	1241 (10.6)
2013	2105 (11.1)	570 (11.9)	296 (12.2)	1239 (10.6)
2014	1884 (10.0)	443 (9.2)	220 (9.1)	1221 (10.4)
2015	1885 (10.0)	400 (8.3)	223 (9.2)	1262 (10.8)
2016	1706 (9.0)	390 (8.1)	235 (9.7)	1081 (9.2)
CGAS case (*n* = 7235)^c^				
No	5889 (81.4)	4057 (84.4)	1832 (75.5)	–
Yes	417 (5.8)	299 (6.2)	118 (4.9)	–
Not reported (missing)	929 (12.8)	452 (9.4)	477 (19.7)	
HoNOS score (*n* = 11,696)^d^				
Subclinical	434 (3.7)	–	–	434 (3.7)
Mild	2033 (17.4)	–	–	2033 (17.4)
Moderately severe	1909 (16.3)	–	–	1909 (16.3)
Very severe	3082 (26.4)	–	–	3082 (26.4)
Not reported (missing)	4238 (36.2)	–	–	4238 (36.2)

Frequencies and proportions were calculated using the original, non-imputed dataset

Values are given as *n*(%); column percentages are reported

*S.D.* standard deviation, *GP* general practice, *CGAS* Children’s Global Assessment Scale, *HoNOS* Health of the Nation Outcome Scales

^a^Secondary health services include Accident and Emergency, clinical specialties, and mental health trusts

^b^Social/criminal justice includes local authority and non-SLaM social services, courts, police, probation services, and youth justice services

^c^12–17 years only

^d^18–29 years only

**Table 2:** Prevalence estimates and relative risk ratios for associations between sociodemographic indicators and referral source in separate models for 12–15, 16–17, and 18–29 age groups

	Referral source						
	GP (reference)^a^	Secondary health services^b^			Social/criminal justice services^c^		
	*n* (%)	*n* (%)	Unadjusted RRR (95% CI)	Adjusted RRR (95% CI)^d^	*n* (%)	Unadjusted RRR (95% CI)	Adjusted RRR (95% CI)^d^
Age 12–15	2227 (69.0)	698 (21.6)	–	–	302 (9.4)	–	–
Ethnicity (*n* = 2872)							
White British	1034 (75.0)	276 (20.0)	1.00	1.00	69 (5.0)	1.00	1.00
White Other	178 (74.8)	45 (18.9)	0.89 (0.64–1.24)	0.79 (0.52–1.19)	15 (6.3)	1.32 (0.73–2.39)	1.06 (0.55–2.05)
Black Caribbean	146 (64.6)	45 (19.9)	1.03 (0.69–1.53)	0.96 (0.64–1.45)	35 (15.5)	3.15 (2.07–4.77)	2.87 (1.88–4.37)
Black African	93 (43.5)	69 (32.2)	2.20 (1.59–3.03)	1.89 (1.27–2.81)	52 (24.3)	6.30 (4.03–9.83)	5.06 (2.95–8.67)
Black British	131 (63.6)	49 (23.8)	1.34 (0.97–1.85)	1.40 (1.02–1.94)	26 (12.6)	3.40 (2.13–5.41)	3.67 (2.31–5.84)
Asian	85 (60.7)	39 (27.9)	1.51 (1.05–2.16)	1.39 (0.95–2.04)	16 (11.4)	2.08 (1.15–3.78)	1.61 (0.86–3.01)
Mixed	218 (69.0)	57 (18.0)	0.92 (0.68–1.24)	0.87 (0.64–1.19)	41 (13.0)	2.65 (1.76–3.99)	2.72 (1.75–4.24)
Other	89 (58.2)	41 (26.8)	1.21 (0.84–1.75)	1.06 (0.66–1.70)	23 (15.0)	2.28 (1.40–3.74)	1.65 (0.90–3.02)
Migration status (*n* = 989)							
Non-migrant	504 (62.2)	243 (30.0)	1.00	1.00	63 (7.8)	1.00	1.00
Migrant	81 (45.3)	67 (37.4)	1.46 (1.07–2.01)	1.32 (0.84–2.07)	31 (17.3)	2.21 (1.36–3.58)	1.32 (0.68–2.56)
Gender (*n* = 3227)							
Female	1367 (69.3)	458 (23.2)	1.00	1.00	149 (7.6)	1.00	1.00
Male	675 (70.9)	148 (15.6)	0.67 (0.55–0.83)	0.66 (0.53–0.81)	129 (13.6)	1.99 (1.62–2.45)	2.14 (1.71–2.66)
Age 16–17	1032 (55.5)	626 (33.6)	–	–	203 (10.9)	–	–
Ethnicity (*n* = 1659)							
White British	449 (62.4)	236 (32.8)	1.00	1.00	35 (4.9)	1.00	1.00
White Other	91 (61.5)	39 (26.4)	0.79 (0.55–1.15)	0.77 (0.49–1.21)	18 (12.2)	2.31 (1.31–4.08)	1.72 (0.76–3.86)
Black Caribbean	51 (43.6)	45 (38.5)	1.42 (0.96–2.10)	1.39 (0.92–2.08)	21 (18.0)	4.49 (2.48–8.15)	4.41 (2.27–8.57)
Black African	57 (33.3)	77 (45.0)	2.11 (1.51–2.93)	2.01 (1.31–3.08)	37 (21.6)	7.17 (4.26–12.05)	5.68 (2.72–11.85)
Black British	36 (38.3)	37 (39.4)	1.73 (1.12–2.65)	1.81 (1.17–2.80)	21 (22.3)	6.44 (3.58–11.57)	7.32 (3.88–13.80)
Asian	60 (46.5)	49 (38.0)	1.51 (1.03–2.20)	1.52 (0.97–2.38)	20 (15.5)	3.68 (2.06–6.59)	2.52 (1.24–5.10)
Mixed	74 (55.2)	42 (31.3)	1.01 (0.66–1.51)	1.02 (0.66–1.58)	18 (13.4)	2.78 (1.51–5.12)	3.01 (1.52–5.95)
Other	76 (52.1)	53 (36.3)	1.11 (0.78–1.58)	1.09 (0.68–1.76)	17 (11.6)	2.37 (1.33–4.22)	1.34 (0.56–3.22)
Migration status (*n* = 744)							
Non-migrant	200 (37.2)	299 (55.6)	1.00	1.00	39 (7.3)	1.00	1.00
Migrant	70 (34.0)	105 (51.0)	0.99 (0.68–1.47)	1.08 (0.73–1.58)	31 (15.1)	2.31 (1.60–3.35)	1.11 (0.55–2.24)
Gender (*n* = 1861)							
Female	685 (56.1)	447 (36.6)	1.00	1.00	89 (7.3)	1.00	1.00
Male	347 (54.2)	179 (28.0)	0.75 (0.61–0.93)	0.75 (0.60–0.93)	114 (17.8)	2.42 (1.78–3.30)	2.60 (1.84–3.67)
Age 18–29	4132 (43.7)	5063 (53.5)	–	–	266 (2.8)	–	–
Ethnicity (*n* = 8631)							
White British	1671 (40.8)	2322 (56.7)	1.00	1.00	102 (2.5)	1.00	1.00
White Other	341 (35.4)	543 (61.3)	1.24 (1.08–1.42)	1.05 (0.84–1.31)	29 (3.3)	1.57 (1.04–2.37)	1.57 (0.90–2.75)
Black Caribbean	173 (41.6)	227 (54.6)	0.94 (0.77–1.13)	0.88 (0.72–1.08)	16 (3.9)	2.79 (1.08–2.96)	2.06 (1.23–3.43)
Black African	256 (32.2)	498 (62.6)	1.48 (1.26–1.75)	1.30 (1.04–1.63)	42 (5.3)	3.28 (2.23–4.81)	3.67 (2.28–5.90)
Black British	75 (25.7)	199 (68.2)	1.73 (1.36–2.20)	1.78 (1.39–2.28)	18 (6.2)	3.96 (2.34–6.70)	4.63 (2.68–7.99)
Asian	226 (33.7)	433 (64.6)	1.41 (1.21–1.65)	1.19 (0.97–1.47)	11 (1.6)	0.95 (0.46–1.97)	0.91 (0.45–1.84)
Mixed	119 (44.4)	139 (51.9)	0.80 (0.63–1.01)	0.75 (0.59–0.96)	10 (3.7)	1.48 (0.75–2.92)	1.60 (0.80–3.22)
Other	763 (63.2)	427 (35.4)	0.48 (0.43–0.55)	0.39 (0.32–0.49)	18 (1.5)	0.49 (0.30–0.81)	0.48 (0.25–0.94)
Migration status (*n* = 5384)							
Non-migrant	894 (25.4)	2514 (71.5)	1.00	1.00	110 (3.1)	1.00	1.00
Migrant	483 (25.9)	1322 (70.9)	1.03 (0.93–1.14)	1.18 (0.96–1.44)	61 (3.3)	1.13 (0.83–1.54)	0.90 (0.54–1.48)
Gender (*n* = 9461)							
Female	2554 (44.6)	3086 (53.9)	1.00	1.00	91 (1.6)	1.00	1.00
Male	1578 (42.3)	1977 (53.0)	1.02 (0.93–1.11)	1.03 (0.93–1.13)	175 (4.7)	2.88 (2.23–3.72)	3.14 (2.41–4.10)

Frequencies and proportions were calculated using the original, non-imputed dataset. Relative risk ratios were calculated using the imputed dataset

^a^GP, general practice, is the reference group

^b^Secondary health services include A&E, other clinical speciality, and other mental health trust

^c^Social/criminal justice includes local authority and non-SLaM social services, courts, police, probation services, and youth justice services

^d^Models adjusted for ethnicity, migration status, gender, household composition, year of referral, and CGAS score (continuous) for those aged 12–15 and 16–17 or HoNOS score (continuous) for those aged 18–29

**Table 3:** Prevalence estimates and relative risk ratios for associations between sociodemographic indicators and referral destination in separate models for 12–15, 16–17, and 18–29 age groups

	Referral destination						
	Outpatient (reference)^a^	Inpatient			Emergency^b^		
	*n* (%)	*n* (%)	Unadjusted RRR (95% CI)	Adjusted RRR (95% CI)^c^	*n* (%)	Unadjusted RRR (95% CI)	Adjusted RRR (95% CI)^c^
Age 12–15	4233 (88.0)	257 (5.3)	–	–	214 (4.5)	–	–
Ethnicity (*n* = 4222)							
White British	1759 (90.0)	110 (5.6)	1.00	1.00	86 (4.4)	1.00	1.00
White Other	299 (90.3)	22 (6.7)	1.17 (0.73–1.90)	1.33 (0.78–2.28)	10 (3.0)	0.69 (0.36–1.31)	0.72 (0.36–1.41)
Black Caribbean	327 (90.1)	26 (7.2)	1.30 (0.83–2.04)	1.31 (0.82–2.09)	10 (2.8)	0.58 (0.31–1.12)	0.60 (0.31–1.17)
Black African	294 (83.1)	29 (8.2)	1.58 (1.03–2.43)	1.76 (1.07–2.91)	31 (8.8)	1.92 (1.26–2.94)	1.99 (1.23–3.24)
Black British	272 (91.0)	9 (3.0)	0.54 (0.27–1.08)	0.57 (0.29–1.15)	18 (6.0)	1.26 (0.73–2.17)	1.36 (0.79–2.34)
Asian	185 (86.1)	20 (9.3)	1.68 (1.02–2.77)	1.85 (1.08–3.17)	10 (4.7)	0.99 (0.50–1.97)	1.03 (0.51–2.08)
Mixed	427 (91.0)	27 (5.8)	1.01 (0.65–1.57)	1.02 (0.66–1.60)	15 (3.2)	0.68 (0.39–1.18)	0.64 (0.37–1.12)
Other	203 (86.0)	11 (4.7)	0.81 (0.43–1.53)	0.93 (0.46–1.88)	22 (9.3)	1.82 (1.12–2.95)	2.14 (1.19–3.83)
Migration status (*n* = 1429)							
Non-migrant	937 (79.6)	125 (10.6)	1.00	1.00	115 (9.8)	1.00	1.00
Migrant	196 (77.8)	27 (10.7)	1.17 (0.83–1.66)	0.86 (0.55–1.34)	29 (11.5)	1.13 (0.72–1.77)	0.87 (0.48–1.57)
Gender (*n* = 4704)							
Female	2734 (89.0)	167 (5.4)	1.00	1.00	170 (5.5)	1.00	1.00
Male	1499 (91.8)	90 (5.5)	0.98 (0.76–1.28)	0.92 (0.70–1.21)	44 (2.7)	0.50 (0.37–0.70)	0.53 (0.38–0.74)
Age 16–17	1817 (74.9)	188 (7.7)	–	–	361 (14.9)	–	–
Ethnicity (*n* = 2107)							
White British	666 (75.1)	81 (9.1)	1.00	1.00	140 (15.8)	1.00	1.00
White Other	158 (81.4)	12 (6.2)	0.66 (0.35–1.23)	0.58 (0.27–1.28)	24 (12.4)	0.77 (0.48–1.23)	1.05 (0.63–1.76)
Black Caribbean	126 (75.9)	15 (9.0)	1.04 (0.58–1.88)	1.06 (0.56–2.01)	25 (15.1)	0.97 (0.61–1.55)	1.09 (0.68–1.77)
Black African	156 (69.3)	19 (8.4)	1.05 (0.62–1.78)	0.92 (0.46–1.87)	50 (22.2)	1.53 (1.06–2.21)	2.10 (1.37–3.22)
Black British	95 (75.4)	11 (8.7)	1.01 (0.52–1.97)	1.01 (0.50–1.99)	20 (15.9)	1.05 (0.63–1.75)	1.08 (0.64–1.82)
Asian	112 (67.9)	21 (12.7)	1.63 (0.97–2.73)	1.48 (0.76–2.88)	32 (19.4)	1.39 (0.90–2.14)	1.92 (1.20–3.09)
Mixed	124 (77.5)	10 (6.3)	0.67 (0.34–1.32)	0.65 (0.32–1.31)	26 (16.3)	0.94 (0.59–1.51)	0.99 (0.62–1.58)
Other	134 (72.8)	18 (9.8)	1.11 (0.65–1.90)	0.99 (0.48–2.08)	32 (17.4)	1.10 (0.72–1.67)	1.65 (1.01–2.68)
Migration status (*n* = 928)							
Non-migrant	354 (53.4)	93 (14.0)	1.00	1.00	216 (32.6)	1.00	1.00
Migrant	168 (63.4)	35 (13.2)	1.14 (0.78–1.68)	1.08 (0.58–2.02)	62 (23.4)	0.84 (0.63–1.12)	0.56 (0.38–0.82)
Gender (*n* = 2366)							
Female	1161 (76.5)	100 (6.6)	1.00	1.00	256 (16.9)	1.00	1.00
Male	656 (77.3)	88 (10.4)	1.56 (1.15–2.11)	1.45 (1.06–2.00)	105 (12.4)	0.73 (0.57–0.94)	0.73 (0.56–0.94)
Age 18–29	6176 (52.8)	1030 (8.8)	–	–	4170 (35.7)	–	
Ethnicity (*n* = 10331)							
White British	2491 (51.1)	448 (9.2)	1.00	1.00	1937 (39.7)	1.00	1.00
White Other	484 (46.5)	93 (8.9)	1.06 (0.83–1.36)	0.98 (0.70–1.36)	464 (44.6)	1.24 (1.07–1.43)	1.26 (1.04–1.53)
Black Caribbean	255 (47.9)	79 (14.9)	1.70 (1.30–2.23)	1.60 (1.19–2.15)	198 (37.2)	1.02 (0.84–1.23)	1.05 (0.86–1.28)
Black African	399 (40.9)	159 (16.3)	2.23 (1.81–2.76)	1.99 (1.50–2.65)	417 (42.8)	1.39 (1.20–1.62)	1.44 (1.18–1.75)
Black British	126 (40.0)	30 (8.8)	1.29 (0.86–1.94)	1.31 (0.86–1.99)	185 (54.3)	1.87 (1.50–2.34)	1.96 (1.55–2.46)
Asian	364 (45.6)	72 (9.0)	1.11 (0.84–1.46)	1.01 (0.73–1.39)	363 (45.4)	1.33 (1.13–1.57)	1.30 (1.06–1.59)
Mixed	175 (52.4)	40 (12.0)	1.21 (0.85–1.74)	1.15 (0.79–1.67)	119 (35.6)	0.86 (0.67–1.10)	0.85 (0.66–1.10)
Other	1015 (70.8)	100 (7.0)	0.57 (0.45–0.71)	0.51 (0.38–0.69)	318 (22.2)	0.43 (0.37–0.50)	0.42 (0.34–0.51)
Migration status (*n* = 6300)							
Non-migrant	1455 (35.2)	370 (8.9)	1.00	1.00	2313 (55.9)	1.00	1.00
Migrant	789 (36.5)	238 (11.0)	1.10 (0.94–1.28)	1.11 (0.86–1.42)	1135 (52.5)	0.92 (0.82–1.03)	0.95 (0.79–1.13)
Gender (*n* = 11376)							
Female	3938 (57.0)	529 (7.7)	1.00	1.00	2448 (35.4)	1.00	1.00
Male	2238 (50.2)	501 (11.2)	1.66 (1.46–1.90)	1.61 (1.40–1.85)	1722 (38.6)	1.25 (1.15–1.35)	1.26 (1.16–1.37)

Frequencies and proportions were calculated using the original, non-imputed dataset. Relative risk ratios were calculated using the imputed dataset

^a^Outpatient services is the reference group

^b^Emergency services include A&E Liaison Croydon University Hospital, Criminal Justice Mental Health Services, Croydon 136 Suite, Mental Health Liaison Service at King’s College Hospital, Lewisham 136 Suite, Liaison Psychiatry at King’s College Hospital, Liaison Psychiatry at St. Thomas’ Hospital, Place of Safety, St. Thomas’ Hospital A&E Services

^c^Models adjusted for ethnicity, migration status, gender, household composition, year of referral, and CGAS score (continuous) for those aged 12–15 and 16–17 or HoNOS score (continuous) for those aged 18–29

**Table 4:** Prevalence estimates and odds ratios for associations between referral source and referral location in separate models for 12**–**15, 16**–**17, and 18**–**29 age groups

	Referral location			
	Outpatient (reference)^a^	Inpatient		
	*n* (%)	*n* (%)	Unadjusted OR (95% CI)	Adjusted OR(95% CI)^d^
Age 12–15 (*n* = 4490)	4233 (94.3)	257 (5.7)	–	–
Referral source (*n* = 2978)				
GP	2096 (95.3)	103 (4.7)	1.00	1.00
Secondary health services^b^	442 (91.0)	44 (9.1)	2.13 (1.52–2.98)	1.85 (1.28–2.68)
Social/legal services^c^	278 (94.9)	15 (5.1)	1.51 (0.91–2.49)	1.29 (0.73–2.29)
Ethnicity (*n* = 4020)				
White British	1759 (94.1)	110 (5.9)	1.00	1.00
White Other	299 (93.2)	22 (6.8)	1.18 (0.74–1.90)	1.39 (0.81–2.40)
Black Caribbean	327 (92.6)	26 (7.4)	1.31 (0.84–2.05)	1.29 (0.81–2.06)
Black African	294 (91.0)	29 (9.0)	1.61 (1.05–2.46)	1.70 (1.01–2.85)
Black British	272 (96.8)	9 (3.2)	0.54 (0.27–1.07)	0.53 (0.26–1.07)
Asian	185 (90.2)	20 (9.8)	1.69 (1.02–2.78)	1.77 (1.02–3.05)
Mixed	427 (94.1)	27 (5.9)	1.03 (0.67–1.60)	1.03 (0.66–1.62)
Other	203 (94.9)	11 (5.1)	0.82 (0.44–1.54)	0.96 (0.48–1.94)
Migration status (*n* = 1285)				
Non-migrant	937 (88.2)	125 (11.8)	1.00	1.00
Migrant	196 (87.9)	27 (12.1)	1.18 (0.83–1.67)	0.82 (0.52–1.30)
Gender (*n* = 4490)				
Female	2734 (94.2)	167 (5.8)	1.00	1.00
Male	1499 (94.3)	90 (5.7)	0.98 (0.75–1.28)	0.93 (0.70–1.23)
Age 16–17 (*n* = 2005)	1817 (90.6)	188 (9.4)	–	–
Referral source (*n* = 1464)				
GP	944 (94.4)	56 (5.6)	1.00	1.00
Secondary health services^b^	225 (82.7)	47 (17.3)	2.92 (1.95–4.37)	2.89 (1.81–4.61)
Social/legal services^c^	177 (92.2)	15 (7.8)	1.53 (0.86–2.71)	1.10 (0.58–2.09)
Ethnicity (*n* = 1758)				
White British	666 (89.2)	81 (10.8)	1.00	1.00
White Other	158 (92.9)	12 (7.1)	0.65 (0.34–1.22)	0.63 (0.28–1.41)
Black Caribbean	126 (89.4)	15 (10.6)	1.03 (0.58–1.85)	1.04 (0.54–2.00)
Black African	156 (89.1)	19 (10.9)	1.03 (0.61–1.76)	0.96 (0.45–2.05)
Black British	95 (89.6)	11 (10.4)	0.99 (0.51–1.93)	0.92 (0.45–1.88)
Asian	112 (84.2)	21 (15.8)	1.61 (0.96–2.71)	1.51 (0.76–3.02)
Mixed	124 (92.5)	10 (7.5)	0.67 (0.33–1.34)	0.62 (0.30–1.30)
Other	134 (88.2)	18 (11.8)	1.10 (0.64–1.89)	1.01 (0.47–2.14)
Migration status (*n* = 650)				
Non-migrant	354 (79.2)	93 (20.8)	1.00	1.00
Migrant	168 (82.8)	35 (17.2)	1.12 (0.77–1.64)	0.97 (0.49–1.92)
Gender (*n* = 2005)				
Female	1161 (92.1)	100 (7.9)	1.00	1.00
Male	656 (88.2)	88 (11.8)	1.56 (1.15–2.11)	1.59 (1.13–2.22)
Age 18–29 (*n* = 7206)	6176 (85.7)	1030 (14.3)	–	–
Referral source (*n* = 5215)				
GP	3456 (88.8)	435 (11.2)	1.00	1.00
Secondary health services^b^	890 (77.3)	262 (22.7)	2.35 (1.99–2.77)	2.42 (2.04–2.87)
Social/legal services^c^	83 (48.3)	89 (51.7)	6.71 (4.86–9.27)	5.06 (3.64–7.05)
Ethnicity (*n* = 6330)				
White British	2491 (84.8)	448 (15.2)	1.00	1.00
White Other	484 (83.9)	93 (16.1)	1.05 (0.82–1.34)	0.99 (0.71–1.41)
Black Caribbean	255 (76.4)	79 (23.6)	1.70 (1.29–2.22)	1.60 (1.17–2.19)
Black African	399 (71.5)	159 (28.5)	2.22 (1.80–2.73)	1.84 (1.32–2.56)
Black British	126 (80.8)	30 (19.2)	1.26 (0.84–1.90)	1.13 (0.73–1.75)
Asian	364 (83.5)	72 (16.5)	1.10 (0.84–1.44)	1.05 (0.74–1.48)
Mixed	175 (81.4)	40 (18.6)	1.21 (0.84–1.73)	1.14 (0.77–1.69)
Other	1015 (91.0)	100 (9.0)	0.56 (0.44–0.70)	0.61 (0.45–0.84)
Migration status (*n* = 2852)				
Non–migrant	1455 (79.7)	370 (20.3)	1.00	1.00
Migrant	789 (76.8)	238 (23.2)	1.09 (0.92–1.28)	1.03 (0.77–1.37)
Gender (*n* = 7206)				
Female	3938 (88.2)	529 (11.8)	1.00	1.00
Male	2238 (81.7)	501 (18.3)	1.67 (1.46–1.90)	1.56 (1.35–1.81)

Frequencies and proportions were calculated using the original, non-imputed dataset. Odds ratios were calculated using the imputed dataset

^a^Outpatient services are the reference group. Emergency services were dropped from the analyses due to low cell sizes

^b^Secondary health services include A&E, other clinical speciality, and other mental health trust

^c^Social/legal includes social services and police/courts

^d^Models adjusted for referral source, ethnicity, migration status, gender, household composition, year of referral, and CGAS score (continuous) for those aged 12–15 and 16–17 or HoNOS score (continuous) for those aged 18–29

## Results

### Sample characteristics

**Fig. 1:**
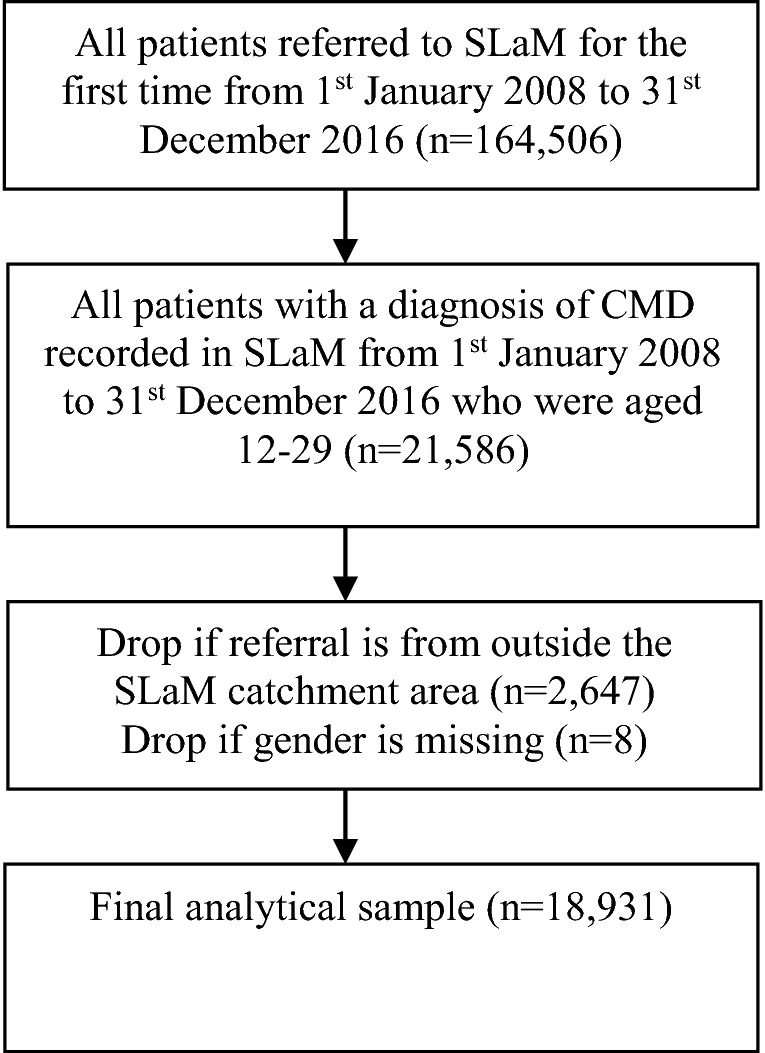
Final analytical sample

21,586 individuals aged 12–29 with a valid diagnosis of anxiety or non-psychotic depressive disorder were identified using the CRIS data extraction tool (see Fig. 1). Referrals of young people from outside the SLaM catchment area to National Specialist teams (*n* = 2647) were excluded due to patients not living in the SLaM catchment area and thus differing from those referred locally in terms of age, gender, migration status, and ethnicity. A further eight patients were excluded due to missing information on gender, one of the key explanatory measures. 18,931 individuals remained in the final analytical sample (see Fig. 1). Due to the limitations of the case register approach, some variables had high levels of missingness which have been reported in Table 1 and corrected with multiple imputation. Similar findings were observed between original and imputed data.

The mean age of the sample was 20.3 years (standard deviatio*n* = 5.3 years). Over half of the sample (62%) was aged 18–29, approximately 25% were aged 12–15, and the remaining 13% were aged 16–17. Just under half the sample identified as White British (42%) ethnicity. The largest ethnic minority groups were Other (not specified) ethnicity (10%), Black African (8%), and White Other (8%). Most of the samples were female (63%). The most common types of referral source were GP and secondary health services, with only 4% of the sample referred from social/criminal justice services. The majority of the samples were referred to outpatient services (65%), approximately 25% of the sample were referred to emergency services, and 8% were referred to inpatient services. Those aged 12–15 tended to be White British, non-migrant, female, living with parents, referred from GP and referred to outpatient services. In contrast, those aged 16–17 had the largest proportion of ethnic minorities and referrals from social/criminal justice services. The oldest age group, 18–29, differed from the youngest age group in that they had a higher proportion of migrants, those referred from secondary health services, and those referred to inpatient and emergency services.

### Inequalities in referral source

#### Inequalities in referrals from secondary health services

There were notable differences in referral source by ethnicity and migration status in separate models for each age group (Table 2). In unadjusted models, those who identified as Black African ethnicity had a two times increase in the likelihood of being referred from secondary health services compared to GP than the White British group in the 12–15 and 16–17 age groups. Among those in the 18–29 age group, those who identified as Black African, Black British, and Asian were more likely than the White British ethnic group to be referred from secondary health services compared to GP. Conversely, Other ethnicity was associated with decreased likelihood of referral from secondary health services compared to GP. In the 12–15 and 16–17 age groups, males were less likely than females to be referred from secondary health services compared to GP. These associations remained significant following adjustment for migration status, gender, household composition, year of referral, and psychosocial functioning. It was only in the 12–15 age group that migrants were more likely than non-migrants to be referred from secondary health services compared to GP. However, this association was attenuated in the adjusted model.

#### Inequalities in referrals from social/criminal justice services

Across all age groups, most ethnic minority groups compared to the White British group had a two-to-sixfold increase in the likelihood of being referred from social/criminal justice services compared to GP in support of our first hypothesis (Table 2). This difference was the largest among the Black ethnic groups in the group aged 16–17, even after adjusting for level of mental health need and other potential confounders. Migrants in groups aged 12–15 and 16–17 were more likely than non-migrants to be referred from social/criminal justice services compared to GP in unadjusted models, but the effect of migration status was attenuated in the fully adjusted model. Notably, males in each age group had at least two times increase in the likelihood of being referred from social/criminal justice services compared to GP than female patients in adjusted models.

### Inequalities in referral destination

#### Inequalities in referrals to inpatient services

The identified differences in referral destination by ethnicity varied across the age groups (Table 3). Among those in the 12–15 age group, those in the Black African and Asian ethnic groups were more likely than White British individuals to be referred to inpatient services compared to outpatient services as the reference. This difference was also present for the Black African group in the 18–29 age group along with the Black Caribbean ethnic group, but not among the Asian group. Notably, there were no differences in inpatient services by ethnicity in the 16–17 age group. Conversely, Other ethnicity was associated with decreased likelihood of referral to inpatient services compared to outpatient services in the 18–29 age group. There were no identified differences by migration status in any of the age groups. For gender, males were more likely than females to be referred to inpatient services compared to outpatient services in the 16–17 and 18–29 age groups but not in the 12–15 age group.

#### Inequalities in referrals to emergency services

For emergency services, those in the Black African ethnic group were more likely than the White British group be directed to this referral destination in all three age groups. This was also the case in adjusted models for those categorised as Other ethnicity in the 12–15 age group, those in the Asian ethnic group in the 16–17 age group, and the Black British and Asian ethnic groups in the 18–29 age group. These findings support our second hypothesis. In contrast, for those aged 18–29, Other ethnicity was associated with being less likely to be referred to emergency services in adjusted models which did not support our second hypothesis. Differences by migration status were only present among migrants in the 16–17 age group who were less likely to be referred to emergency services compared to the reference. Interestingly, males were less likely than females to be referred to emergency services compared to outpatient services in the two younger age groups, but more likely to be referred to emergency services in the 18–29 age group.

### Associations between referral source and referral destination

Emergency services were dropped from the analyses due to low cell sizes when stratified by referral source; it is highly unlikely for a patient to be referred to emergency services from GP (*n* = 12, 0.1%) or social/legal services (*n* = 109, 0.6%). Therefore, Table 4 shows the likelihood of being referred to inpatient services compared to outpatient services (reference group). Across the three age groups, those referred from secondary health services were significantly more likely than those referred from GP to be referred to inpatient services compared to outpatient services, with over twice the odds for those aged 16–17 and 18–29 (Table 4). In the oldest age group only, those referred from social/criminal justice services were significantly more likely than those referred from GP to be referred to inpatient services compared to outpatient services in both unadjusted and adjusted models. In adjusted analyses for the 12–15 age group, those in the Black African and Asian ethnic groups were more likely than the White British group to be referred to inpatient services compared to outpatient services. There were no significant differences between ethnic groups in the 16–17 group; however, males were more likely than females to be referred to inpatient services compared to outpatient services in adjusted analyses. In the 18–29 age group, there were significant differences by gender and ethnicity in adjusted analyses, with males and those who identified as Black Caribbean and Black African ethnicity being more likely than females and White British individuals, respectively, to be referred to inpatient services compared to outpatient services. There were no identified differences by migration status in any of the age groups.

## Discussion

Ethnicity has a significant role in determining how young people receive mental health care. Using data from electronic mental healthcare records, the present study examined inequalities in both referral source and referral destination for young people aged 12–29 accessing secondary mental health services in south east London. We identified 18,931 individuals within this age bracket with a diagnosis of anxiety or non-psychotic depressive disorder who were first referred to services between the 1st January 2008 and 31st December 2016. Our analyses showed that Black African individuals were more likely than White British individuals to be referred from secondary health or social/criminal justice services compared to general practice (GP), and the effect was most pronounced for those aged 16–17 years. This finding supports our first hypothesis that ethnic minority patients, especially those aged 16–17, would be more likely than White British patients to be referred from social/criminal justice services compared to GP. Black African individuals were also significantly more likely to be referred to inpatient and emergency services than outpatient services. Regarding the association between referral source and referral destination, we found that those referred from secondary health services were significantly more likely to be referred to inpatient than outpatient services compared to those referred from GP. For those aged 18–29, referral from social/criminal justice services was significantly associated with referral to inpatient services. There were no identified differences by migration status in any of the age groups.

### Comparisons to previous studies

The present study found that those who identified as Black African were more likely than those who identified as White British to be referred from secondary health or social/criminal justice services compared to GP, and the effect was most pronounced for those aged 16–17 years, with a sevenfold and sixfold increase in odds for the Black African and Black British groups, respectively. Our findings are consistent with evidence from a previous study conducted in south east London which found that African–Caribbean patients were significantly less likely to access care through a general practitioner than were White British patients, with less than 30% of African–Caribbean patients accessing care in this way [11]. Similar patterns have been found in young people accessing NHS-provided Child and Adolescent Mental Health Services (CAMHS) [48, 49]. We also found that less than 20% of Black African patients were referred to services by a general practitioner which is in line with the previous studies [11]. Our findings suggest that Black individuals living in south east London, particularly those who identify as Black African, may face barriers to accessing primary care that their White British counterparts do not. For example, previous research conducted with south east London residents suggest that there are ethnic group differences in whether help for mental health problems is sought from friends, family or religious leaders, with evidence for Black African individuals being more likely to seek help from a religious leader and less likely to seek formal help [50]. Further qualitative research with individuals from the same community sample found that reasons for not seeking professional help for mental health problems included perceiving their problems as normal or unsuitable for professional help, negative expectations of professional help, believing informal strategies were sufficient, fearing being stigmatised for having a mental disorder and help-seeking, and self-perceptions of being strong and/or self-reliant [51]. The intensity and nature of stigmatising beliefs about mental illness are affected by cultural beliefs and vary between ethnic minority groups [52]. For example, within some Black communities in the UK, people with mental health problems are seen as violent or dangerous [53]. As a result, families can both stigmatise the person with mental illness, and in turn, themselves be stigmatised through association. Such stigma can act as a barrier to young people seeking help for mental health problems, particularly if they can only access services when taken by a parent/caregiver.

Crucially, our findings show that ethnic variations in referral source are greatest for those aged 16–17 who may already face difficulties accessing mental health services or transitioning from adolescent to adult services [54]. This study highlights the importance of providing support to ethnic minority adolescents accessing secondary mental health care through their GP and ensuring that it is maintained after they turn 18. There remains a lack of robust research available on transitional care for adolescents accessing mental health services. Further research is needed on young people’s journeys through the transition from adolescent services to adult services.

Our results showing that Black African and Black Caribbean individuals were approximately twice as likely as White British individuals to be referred to inpatient services compared to outpatient services support findings from the previous studies which show that ethnic minorities are more likely to be referred to inpatient services than their White counterparts [15, 17]. Conversely, “Other” ethnicity was associated with decreased odds of referral to inpatient services compared to outpatient services. However, it is hard to draw conclusions from this finding as we were unable to discern which ethnicities were included in this category due to insufficient recording of these data in the clinical records.

It was found that Black African individuals in the 16–17 group and Black British individuals in the 18–29 group were more likely than those who identified as White British to be referred to emergency services compared to outpatient services. These findings partially support our second hypothesis that ethnic minority individuals, especially those aged 16–17, would be more likely than White British individuals to be referred to emergency services compared to outpatient services. Our findings are consistent with the existing literature that shows ethnic minorities are more likely than White individuals to use emergency mental health services [15]. Together with the finding that Black African individuals are less likely to access secondary mental health services through their GP and are more likely to be referred by social/criminal justice services, our results suggest that Black African individuals experience more negative pathways to care at both the point of access to secondary mental health services and also once they have been accepted for treatment. Being referred from social/criminal justice services and being referred to emergency services are both characterised by involuntary admission at a point of crisis with severe and long-lasting consequences for the individual. These findings suggest that Black African individuals may not receive the help which they need in time, perhaps because of a reluctance to use mental health services or as a result of other cultural and systemic barriers to care [55, 56], resulting in the worsening of symptoms over time until drastic last-resort measures are taken. Moreover, it has been found that Black African adolescents are less likely to stay in treatment than their White peers, further highlighting the importance of providing culturally appropriate mental health treatments for young people from ethnic minority backgrounds [57–59].

The final set of findings is related to the associations between referral source and referral to inpatient services compared to outpatient services; emergency services were dropped from the analyses due to low cell sizes. The results showed that those referred from secondary health services or social/criminal justice services were significantly more likely than those referred from GP to be referred to inpatient services compared to outpatient services. This result suggests that those who are referred to services via more adverse pathways (i.e., secondary health services and social/criminal justice services compared to GP) may find themselves being referred for high-intensity treatment requiring admission to hospital (i.e., inpatient treatment compared to outpatient treatment). To the best of our knowledge, there are no recent studies conducted in the UK that examine the relationship between referral source and referral to inpatient and outpatient services. Overall, the findings from this study suggest that inequalities in referral destination may be perpetuated by inequalities generated at the point of access.

### Strengths and limitations

In this study, we used an innovative data resource that allowed us to identify a large cohort from an ethnically diverse area and assess differences in referral pathways for each ethnic minority group. Most of the research in this area has been conducted in the US and often lacks the power to look at the differences between Black African and Black Caribbean groups. Using natural language-processing applications to extract detailed information on ethnicity from unstructured or free text, this study was able to identify Black African, Black Caribbean, and Black British individuals. Psychiatric case registers that include complete electronic health records offer exciting opportunities for future research into the referral pathways of young people living in south east London. They do, however, have important limitations: much information of interest is not recorded in a standardised way and the data available, and their quality, depend upon clinicians’ accuracy and timeliness in reporting of clinical information. This is particularly pertinent to the SLAM BRC Case Register, which draws data directly from routine administrative and clinical sources (rather than the older generation of registers which have been more involved in the collection and cleaning of incoming data) [29, 31]. This results in a high proportion of missing data and the need for clinician engagement in the process of generating data of this nature for research purposes. Importantly, migration status was missing for approximately half of the sample. This highlights the need for clinicians to routinely collect this information; however, this may be challenging given the current contentious political and social debates around migration. In addition, referral source was missing for almost a quarter of the sample due to insufficient recording of these data. Further missing data meant that variables used in adjusted models had high proportions of missingness and we were not able to look at the relationship between referral source and admission to emergency services due to low cell sizes. The generalisability of the findings beyond the study setting is unclear and further research in other settings is needed. In different areas of the UK, or indeed different areas of London, mental health services that are available to young people will vary widely, as will the age at which individuals are discharged from child and adolescent services and referred to adult services [3, 60]. Furthermore, the study uses a psychiatric sample of patients whose anxiety and depression symptoms have penetrated through primary care services to secondary mental health care, representing more severe end mental disorder rather than primary care level mental disorder. Therefore, findings could not necessarily be extrapolated to primary care samples only. Nonetheless, this study provides urgently needed evidence on the inequalities in referral pathways for young people accessing secondary mental health services.

### Implications

It is becoming more widely acknowledged that a person’s age does not necessarily dictate need. Although there must be age “cut offs” for services and certain provision, it is important to identify inequalities in health services that could impact the cover provided to young adolescents, especially as they transition to young adulthood. The current system is failing to provide adequate support in childhood/early adolescence when issues may be at their most treatable. The Royal College of General Practitioners recommends that GP trainees in the future should receive specialist-led child health and mental health training, and GP surgeries should aim to be more “youth friendly” by involving young people in patient participation groups, communicating practice news over social media (Twitter and Facebook), and increasing the use of digital technology as a means to connect with their younger patient population. Community organisations such as Black Thrive (www.blackthrive.org.uk), which brings together Lambeth’s Black communities and service providers to reduce the inequality and injustices experienced by Black people in mental health services, need to be ubiquitous in ethnically diverse areas. At an individual level, clinicians need to receive regular implicit bias training to ensure that they do not overlook certain behaviours in some ethnic groups which may be the hallmarks of mental health problems. At a structural level, health inequalities can also be tackled by introducing affirmative action policies to lower the threshold for certain ethnic groups that have a long history of facing barriers to accessing health services.

## Conclusions

This study identifies inequalities in both referral source and referral destination for young people accessing secondary mental health services in south east London. Our findings suggest that there are processes operating prior to first referral that increase the risk of more negative pathways to care for young people from Black British, Black African, and Black Caribbean backgrounds, particularly across the transition between adolescent and adult service provision. The current system for accessing appropriate mental health treatment is inflexible and rigid; it requires young people to fit into services instead of services responding to need, and some fall through the gaps. Further research is needed on young people’s journeys from adolescent to adult services and how inequalities in referral source may impact treatment pathways and patient outcomes.

## Electronic supplementary material

Below is the link to the electronic supplementary material.

Supplementary material 1 (DOCX 12 kb)

## Data Availability

The data that support the findings of this study are available from the National Institute for Health Research (NIHR) Biomedical Research Centre at South London and Maudsley NHS Foundation Trust. Restrictions apply to the availability of these data, which were used under license for this study. Data are available from the authors with the permission of the NIHR Biomedical Research Centre. The custom computer codes used to generate results that are reported in the paper are available from the authors with the permission of the National Institute for Health Research (NIHR) Biomedical Research Centre at South London and Maudsley NHS Foundation Trust.
