# Supplementary material for: Inequalities in referral pathways for young people accessing secondary mental health services in south east London
Source: Eur Child Adolesc Psychiatry. 2020 Jul 18;30(7):1113–28. doi: 10.1007/s00787-020-01603-7 (PMC8295086; doi:10.1007/s00787-020-01603-7)
Supplement: Supplementary file 1 — Supplementary material 1 (DOCX 12 kb) [file 787_2020_1603_MOESM1_ESM.docx]

**Supplementary material**

Supplementary Table 1. Number of missing observations and imputed values in the full sample

|  | **Complete** | **Incomplete** | **Imputed** | **Total** |
| --- | --- | --- | --- | --- |
| Ethnicity | 17,077 | 1,854 | 1,854 | 18,931 |
| Migration status | 8,765 | 10,166 | 10,166 | 18,931 |
| Gender | 18,931 | 0 | 0 | 18,931 |
| Referral source | 14,549 | 4,382 | 4,382 | 18,931 |
| Referral destination | 18,446 | 4,85 | 4,85 | 18,931 |
| Household composition | 10,372 | 8,559 | 8,559 | 18,931 |
| Year of referral | 18,931 | 0 | 0 | 18,931 |
